# IASLC Textbook of Prevention and Early Detection of Lung Cancer

**DOI:** 10.1038/sj.bjc.6603136

**Published:** 2006-05-30

**Authors:** S G Spiro

**Affiliations:** 1 University College of London, London, UK

Perhaps the most important questions in the current management of lung cancer centre around early diagnosis. There has been little tangible progress in the management of established disease, so a book concentrating on prevention and the detection and management of early disease is timely. This is a well-presented, extremely nicely illustrated contribution, edited by four individuals immersed in the early detection and the biology of lung cancer.

The scope of the text ranges through the epidemiology (a fairly conventional chapter), through tobacco and its possible control plus cessation programmes. Then the pathology of early malignant changes and preinvasive lesions are described, together with the biology and proteomics around early disease. These are very up to date, well-written and informative chapters. The value of sputum cytology and then autofluorescence bronchoscopy in early detection are summarised, together with a further chapter on the treatment of carcinoma *in situ* and early invasive disease. A chapter on endobronchial ultrasound in assessment of penetration of disease into the airway walls was new to this reviewer. Seven chapters on screening, based predominantly around low-dose spiral CT, follows. These are good reviews of the principles of screening, the current trials, the evolving nature of CT assessment of intrapulmonary nodules, as well as how to diagnose, follow and treat them.

This book is of limited scope, deliberately so, but provides an extremely well referenced, authoritative review of the subject. References are as up to date as possible for a textbook with a few from 2004. I liked the book, learnt a lot and feel it is a useful addition for any individual treating lung cancer, performing routine bronchoscopy and for pulmonary radiologists as well as basic scientists investigating the biology of this tumour. I can highly recommend it.

